# ﻿*Gelidocalamusalbozonatus* (Poaceae, Bambusoideae), a new species from the southeast of Chongqing, China, and analysis of the morphological diversity in the core group of *Gelidocalamus*

**DOI:** 10.3897/phytokeys.236.111290

**Published:** 2023-11-24

**Authors:** Yong-Long Li, Rong Guo, Hong-Jing Zhang, Si-Rong Yi, Guang-Yao Yang, Wen-Gen Zhang

**Affiliations:** 1 Jiangxi Provincial Key Laboratory for Bamboo Germplasm Resources and Utilization, Forestry College, Jiangxi Agricultural University, Nanchang 330045, China Jiangxi Agricultural University Nanchang China; 2 Collaborative Innovation Center of Jiangxi Typical Trees Cultivation and Utilization, Nanchang 330045, China Collaborative Innovation Center of Jiangxi Typical Trees Cultivation and Utilization Nanchang China; 3 Chongqing Three Gorges Medical College, WanZhou 404120, China Chongqing Three Gorges Medical College WanZhou China

**Keywords:** Arundinarieae, Bambusoideae, leaf epidermis, Poaceae, SEM

## Abstract

*Gelidocalamusalbozonatus* W. G. Zhang, S. R. Yi & Y. L. Li, a new species of *Gelidocalamus*, collected from Pengshui County of Chongqing City in China, was described and illustrated herein. In this study, key morphological characters were compared between the new species and other eight “gelido-” members of *Gelidocalamus*. By using scanning electron microscopy (SEM), its leaf epidermal characters were observed in comparison with those of another three *Gelidocalamus* representatives. Our results show that the new taxon has the typical characteristics of the genus *Gelidocalamus*, both macromorphologically and micromorphologically. Moreover, it was most similar to *G.tessellatus*, but differed by a ring of white tomenta below per node, culm sheath base with densely purple verrucous setae and foliage leaf blades mesophyll.

## ﻿Introduction

*Gelidocalamus* T. H. Wen, is a genus of the temperate woody bamboos (Poaceae, Bambusoideae, Arundinarieae) with only two species (i.e., *G.stellatus* T. H. Wenand *G.tessellatus* T. H. Wen) (Wen 1982). Its typical characteristics include leptomorph rhizomes, each node with many branches (up to 20), terminal branch usually with solitary foliage leaf, semelauctant inflorescence and three stamens. Phenologically, new shoots usually appear in autumn-winter (Wen 1982; Li et al. 2006). However, as the taxon number increased, e.g., nine species in Keng and Wang (1996) and 13 species in Yi et al. (2008), the genus has become more diverse. Particularly, some “spring-shoot” species, e.g., *G.rutilans* T.H Wen, *G.subsolidus* W. T. Lin & Z. J. Feng, *G.solidus* C. D. Chu & C. S. Chao, and *G.longiinternodus* W. T. Wen & S. C. Chen (Wang et al. 2023), expanded the boundary of *Gelidocalamus*.

Recently, Guo et al. (2021) provided a robust phylogenetic tree based on double digest restriction-site associated DNA (ddRAD) sequences of the tribe Arundinarieae in consistence with the analysis of morphological data, revealed that six “gelido-” members (i.e. core members of shooting in autumn and winter) of *Gelidocalamus* were clustered into a monophyletic clade, while other “spring-shoot” members were scattered and grouped with other genera. The “gelido-” taxa have identical micromorphological characters (i.e., prominent stomata apparatus surrounded by 8–12 short papillae and a dense waxy covering) (Wang et al. 2023), including *G.stellatus* T. H. Wen, *G.tessellatus* T. H. Wen & C. C. Chang, *G.annulatus* T. H. Wen,*G.latifolius* Q. H. Dai & T. Chen, *G.multifolius* B. M. Yang, and *G.monophyllus* (Yi et B. M. Yang) B. M. Yang, as well as three recently reported species, i.e. *G.xunwuensis* W. G. Zhang & G. Y. Yang (Zhang et al. 2017), *G.fengkaiensis* N. H. Xia & Z. Y. Cai (Cai et al. 2021), and *G.zixingensis* W. G. Zhang, G. Y. Yang & C. K. Wang (Wang et al. 2023). Moreover, except for *G.monophyllus* (a taxon of the high-elevation distribution, ca. 1250 m), all above taxa were distributed below 1000 m (Li et al. 2016), and have similarly micromorphological characteristics of foliage leaf epidermis, i.e., prominent stomata apparatuses covered with dense wax, and surrounded by 8 to 12 short papillae (Wu et al. 2014; Long et al. 2015; Liu et al. 2017; Nie et al. 2018; Wang et al. 2023).

During a botanical expedition in the southwest of China in 2019, a distinctive “*Gelidocalamus*-like” collection with many branches per node and solitary foliage leaf on each ultimate branch was found from the Wu-Ling Mountain of Chongqing. Then, a complete morphological characterization, including scanning electron microscope (SEM) images of the abaxial leaf epidermis, had been done, and its key features have been also compared with these of other allied species of *Gelidocalamus*. By all the evidence obtained, we believe that this collection is a new species, herein formally described and illustrated.

## ﻿Materials and methods

In the study, eight “gelido-” taxa of *Gelidocalamus* were selected (see Table 1 in detail), and morphologically compared with each other. Key morphological characters, e.g., bamboo shoot, culm and culm leaf, branch and foliage leaf, were surveyed and photographed by DSLR camera (Canon, EOS 60D) with microscope lens (Canon, EF 60mm f/2.8 USM). By using Origin 2021 (OriginLab 2021), foliage leaf blade size has been measured and analyzed based on 30 randomly selected blades of each species, and foliage leaf blade shape of each representative has been drawn based on herbarium specimens. SEM: After washing by using ultrasonic cleaner BRANSON 2800, the middle portion (ca. 5mm × 5mm) of foliage leaf blades was dried at room temperature, mounted on stubs, then sputter-coated with gold powder (3 nm), and observed by using Hitachi S-4800 or Nova NanoSEM 450. Terminologies of the epidermis appendages follows Ellis (1979), Zhang et al. (2014), and Leandro et al. (2019). All voucher specimens were deposited at the herbarium of the College of Forestry, Jiangxi Agricultural University, China (JXAU).

**Table 1.** Voucher information of eight species in the study.

Species	Voucher information
*G.albozonatus*	Pengshui County, Chongqing, China, 108°13'42"N, 29°18'55"E, alt 268 m, *S.R. Yi et al. CQPS01* (JXAU!)
*G.annulatus*	Chishui City, Guizhou, China, 105°95'80"N, 28°47'61"E, alt 809 m, *W.G. Zhang et al. 20151122001* (JXAU!)
*G.latifolius*	Rongshui County, Guangxi, China, 109°10'44"N, 25°13'52"E, alt 229 m, *W.J. Li & Y.G. Liu RS203* (JXAU!)
*G.monophyllus*	Ningyuan County, Hunan, China, 111°98'80"N, 25°23'77"E, alt 1200 m, *W.G. Zhang et al. 20161023* (JXAU!)
*G.multifolius*	Ningyuan County, Hunan, China, 111°57'44"N, 25°19'40"E, alt 346 m, *W.G. Zhang et al. JYS026* (JXAU!)
*G.stellatus*	Jinggangshan City, Jiangxi, China, 114°11'32"N, 26°31'48"E, alt 468 m, *W.G. Zhang et al. JGS003* (JXAU!)
*G.tessellatus*	Libo City, Guizhou, China, 108°07'04"N, 25°20'58"E, alt 526 m, *W.G. Zhang et al. SJJ033* (JXAU!)
*G.xunwuensis*	Xunwu County, Jiangxi , China, 115°28'02"N, 24°54'01"E, alt 526 m, W.G. Zhang et al. 1107(JXAU!)

## ﻿Results and discussion

Eight “gelido-” species (Table 1), including *G.albozonatus*, were observed and compared in detail. We found that the genus has quite rich diversity in the morphology of culm node, culm internode, culm leaf sheath, and the number of branches (Fig. 3). Among them, the ring of white tomenta below the culm nodes and the number of branches of 5–10 in *G.albozonatus* can be distinguished from the other seven taxa. It was also found that *G.albozonatus* resembles *G.tessellatus* most by the number of branches, culm sheath purple patches, and foliage leaf blade size. Thus, it was further found that the new taxa could be distinguished from the latter by glabrous (vs. sparsely setose) internode, densely purple hairs of culm leaf sheath base (vs. smooth), 2–4 pairs of oral setae (vs. hairless), and hairless (vs. densely pubescent) midvein base of foliage leaf (Fig. 4). In a word, based on morphological traits, the above species were easily distinguished (see “Key to nine “gelido-” taxa of the genus *Gelidocalamus*”; Wang et al. 2023).

Interestingly, *Gelidocalamus* showed a rich diversity in terms of foliage leaf blade size and shape (Fig. 5). Based on foliage leaf blade size, eight taxa of the genus can be categorized into three types (Ellis et al. 2009), i.e. mesophyll (including *G.albozonatus*, *G.latifolius* and *G.multifolius*), notophyll (including *G.annulatus*, *G.stellatus*, *G.tessellatus* and *G.xunwuensis*), and microphyll (only *G.monophyllus*). Furthermore, based on foliage leaf blade shape, these taxa also can be divided into two types, i.e. lanceolate (including *G.annulatus*, *G.monophyllus*, *G.stellatus* and *G.xunwuensis*) and elliptic-lanceolate (including *G.albozonatus*, *G.latifolius*, *G.multifolius* and *G.tessellatus*). Thus, it was obvious that foliage leaf blade of *G.albozonatus* was the largest, belonging to the type Mesophyll usually between 77 cm^2^ and 153 cm^2^.

Besides, to reveal the properties of *G.albozonatus* in terms of micromorphological characteristics, its abaxial leaf epidermis was observed by SEM together with three other species (Fig. 6), and referring to previous research (Wu et al. 2014; Zhang et al. 2014; Long et al. 2015; Liu et al. 2017; Zhang et al. 2017; Nie et al. 2018; Wang et al. 2023). Leaf epidermis characters of *G.albozonatus* was identical to that of *G.tessellatus* (Fig. 6B) and *G.annulatus* (Fig. 6C): short papillae scattering on the leaf vein and stomatal zone; stomatal apparatus (usually in 5 to 6 rows between the veins) exposed, surrounded by 8–10 short papillae, but not covered with wax; two types of trichomes (i.e., microhairs and prickles) on the abaxial leaf epidermis, but both sparsely distributed on the abaxial leaf epidermis; silica bodies saddle-shaped, distributed on the veins (Table 2).

**Table 2.** Micromorphology of the abaxial leaf epidermis examined in the present study.

Taxon	Main features	Plates
*G.albozonatus*	prickles, sparse; 8–10 short papillae without dense wax, around the stomata; stomata visible, usually in 5 to 6 rows between the veins	Fig. 2A
*G.tessellatus*	prickles, sparse; 8–10 short papillae with dense wax, around the stomata; stomata visible, usually in 3 rows between the veins	Fig. 2B
*G.annulatus*	prickles, dense; 8–10 short papillae with dense wax, around the stomata; stomata visible, usually in 3 rows between the veins	Fig. 2C
*G.monophyllus*	prickles, dense; short papillae with dense wax; stomata invisible, usually in 3 rows between the veins, totally covered by papillae	Fig. 2D

Currently, *G.albozonatus* is the northernmost distributed species of *Gelidocalamus*, so its discovery has updated the northward distribution line of the genus *Gelidocalamus*.

### ﻿Taxonomic treatment

#### ﻿ Gelidocalamus albozonatus

Taxon classificationPlantaePoalesPoaceae

W.G.Zhang, S.R.Yi & Y.L.Li sp. nov.

7162BE40-6697-5E37-AF2E-7174EED4958C

urn:lsid:ipni.org:names:77331500-1

Figs 1 , 2

##### Type.

China, Chongqing, Pengshui County, Luduhu Village, under the forest, 29°18′55.38″N, 108°13′42.14″E, elev. ca. 268 m, 6 Mar. 2019, *S.R. Yi et al. CQPS01* (holotype: JXAU!).

**Figure 1.**
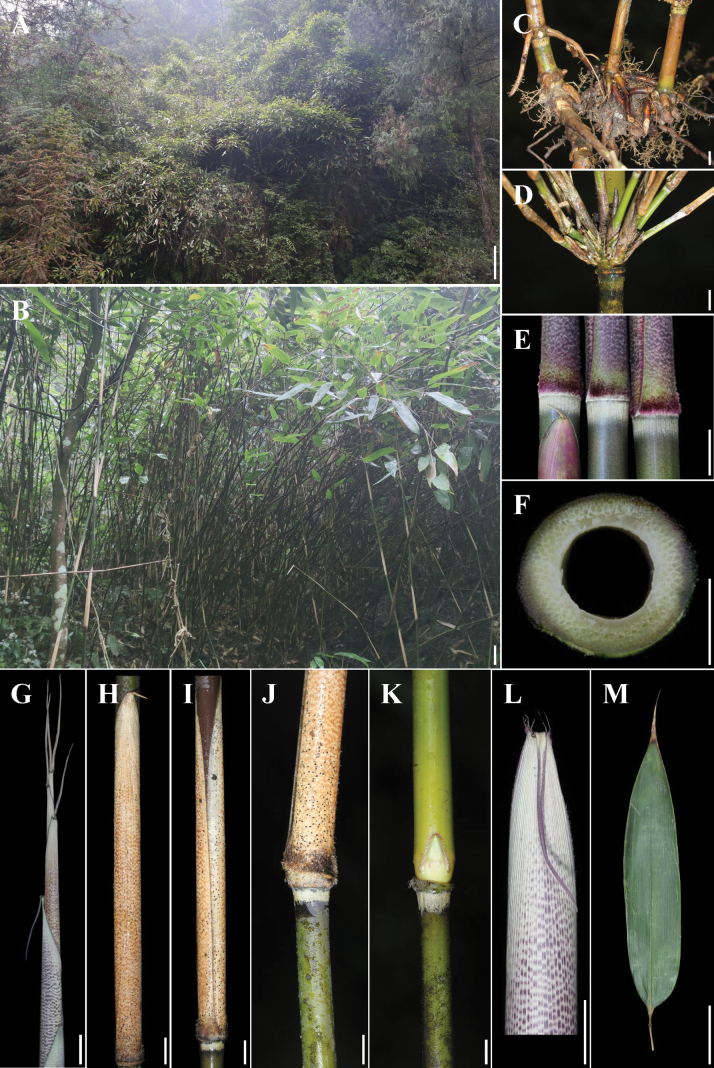
*Gelidocalamusalbozonatus* sp. nov. **A** habitat **B** individual **C** rhizome **D** branches **E** culm node of new shoots **F** transection of culm and pith-cavity **G** dry new shoot **H–L** culms and culm leaves **M** foliage leaf blade. Scale bars: 1 m (**A**); 10 cm (**B**); 1 cm (**C–E, G–L**); 5 mm (**F**); 5 cm (**M**).

##### Diagnosis.

*G.albozonatus* is similar to *G.tessellatus*, but differed by having a ring of white (vs. brownish) tomenta below each culm node, culm sheath base densely purple setulose (vs. yellowish pubescence) and foliage leaf blades mesophyll (vs. notophyll).

**Figure 2.**
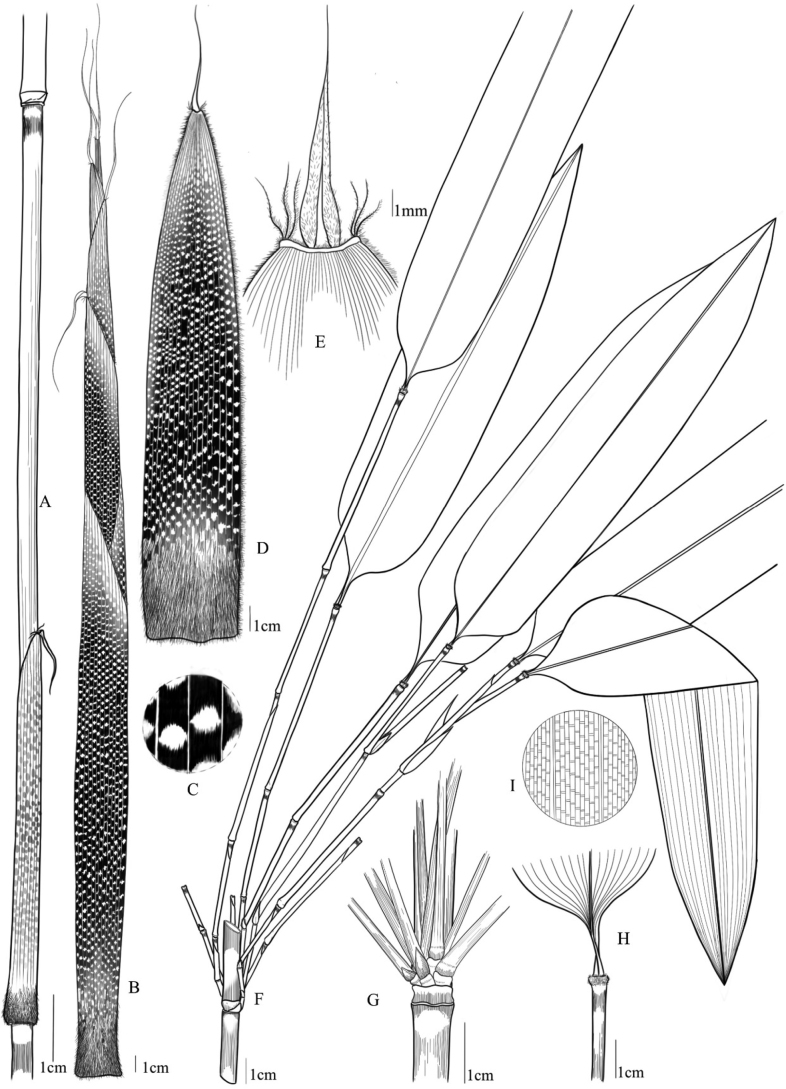
Illustration of *Gelidocalamusalbozonatus***A** culm and culm sheath **B** new shoot **C** details of culm sheath **D** culm leaf **E** oral setae details of culm leaf **F–G** branches and foliage leaves H details of foliage leaf sheath. Drawn by Rong Guo.

##### Description.

Rhizomes leptomorph. Culms up to 5.5 m tall, ca. 6–15 mm in diam., erect, apically slightly nodding; internodes glabrous, 11–54 cm long, wall 1.5–3 mm thick; a ring of white tomenta below each node. Branching intravaginal, arising from the 6^th^ node above ground, ca. 5–10 branches per node; branches equal or subequal, ca. 25–55 cm long, 2–4 mm in diam. Culm leaf sheaths tardily deciduous, 15–25 cm long, abaxially sparsely wine-red or purple hispidulous when young, purple patches densely distributed between transverse veins, sheath base densely purple setulose, ca. 1–3 mm long, margins with wine-red cilia, ca. 1–2 mm long; auricles absent or tiny; oral setae erect or slightly curved, 2–4 pairs, ca. 3–8 mm long; ligule less than 1 mm or absent; blade deciduous, linear or linear-lanceolate, 2–4.5 × 1.7–2.6 mm, erect or recurved, apex acuminate, base constricted with densely short setae, 1/3–1/2 as wide as sheath apex. Ultimate branches usually with one foliage leaf; branch sheath fragile; ligule absent or weak; auricles absent or tiny; blade broadly lanceolate to narrowly oblong, usually 19–42 × 4–7 cm, secondary veins 7–9 pairs, basally cuneate and asymmetrical, abaxially hairless, margins serrulate.

**Figure 3.**
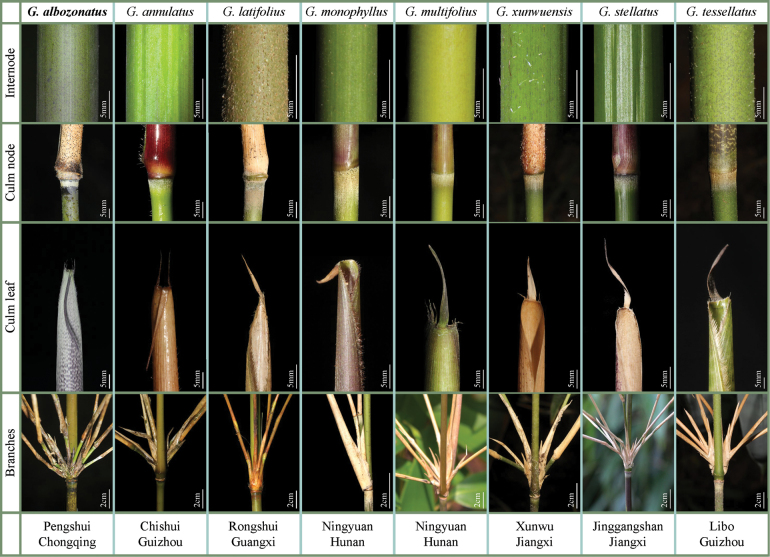
Comparison of key morphological characters between *G.albozonatus* and other seven species. Scale bars: as shown in the figure.

##### Distribution and habitat.

*G.albozonatus* occurs under evergreen broad-leaved forests, along the ravine to the east of Luduhu Village, at elev. ca. 200–600 m. It grows together with *Cupressusfunebris* Endl. (Cupressaceae), *Bambusaemeiensis* L. C. Chia & H. L. Fung (Poaceae), and *Nymphanthuscalcicola* S. R. Yi & Gang Yao, 2022 (Phyllanthaceae), and so on. So far, *G.albozonatus* is only known from one small population (lessthan 1000 m^2^) in the Pengshui County of Chongqing, China.

**Figure 4.**
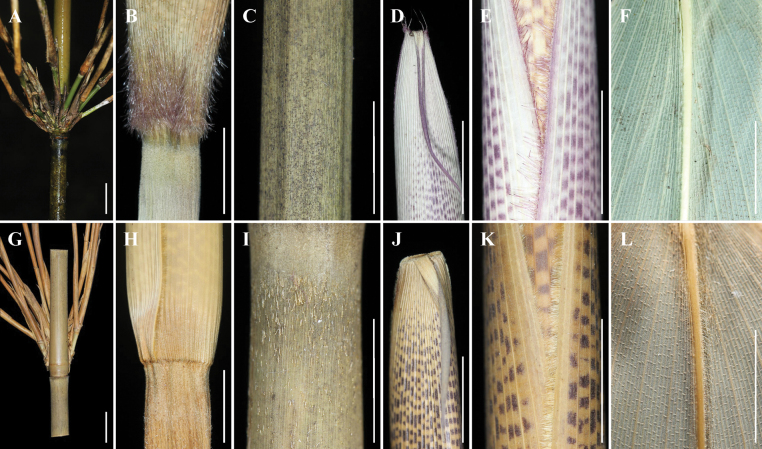
Comparison of morphological characters between *G.albozonatus* (**A–F**) and *G.tessellatus* (**G–L**) **A, G** branches **B, H** clum sheath base **C, I** internode **D, J** oral setae of culm leaf **E, K** margin of clum sheath **F, L** midvein. Scale bars: 1 cm (**A–L**).

##### Chinese vernacular name.

péng-shuǐ-duǎn-zhī-zhú (彭水短枝竹).

##### Phenology.

New shoots Sep–Nov, Inflorescence unknown.

##### Etymology.

The specific epithet indicates the ring of white tomenta below the node.

##### Leaf micromorphology.

Stomatal apparatuses are embossed outwards and smooth without appendages, ca. 27 (25–30) × 13 (11–14) µm. The short papillaes are scattered on the leaf vein and stomatal zone. The exposed stomatal apparatus is surrounded by 8–10 short papillae, but not covered with wax. There are two types of trichomes (i.e., microhairs and prickles) on the abaxial leaf epidermis, but both are sparsely distributed on the abaxial leaf epidermis. The saddle-shaped silica bodies are clearly distributed on the veins. Microhairs are composed of two cells with the apical cell withered, and only occur on the intercostal regions of the abaxial leaf epidermis.

**Figure 5.**
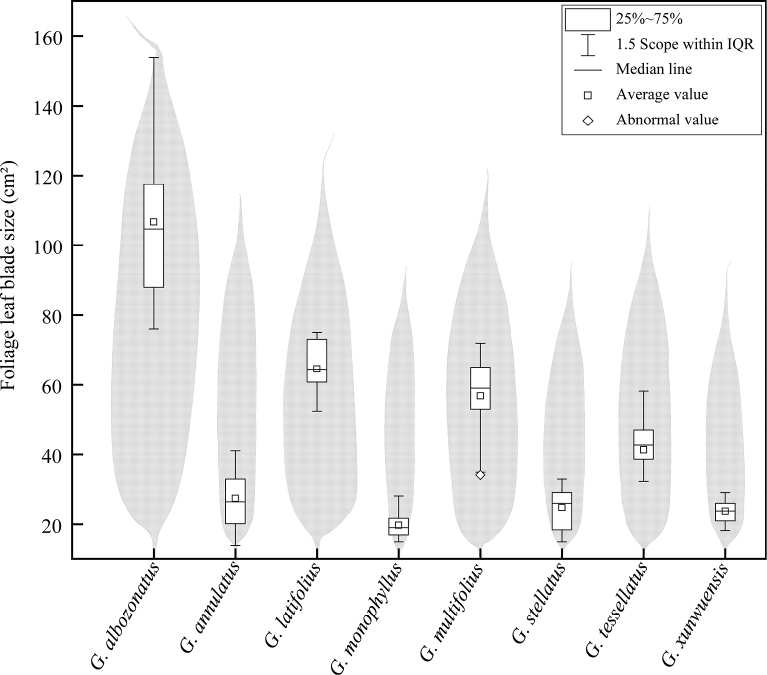
Comparison of foliage leaf blade size and outline between *G.albozonatus* and other seven species.

##### Conservation status.

Based on the field investigations in Pengshui County and adjacent regions (e.g., Shizhu, Qianjiang and Youyang). As the type locality is a mountain with steep terrain, only a population is found on the hillside on both sides of a valley. Therefore, before carrying out further investigations, this species should be assessed as “Data Deficient” (DD), according to the IUCN standards (IUCN 2022).

**Figure 6.**
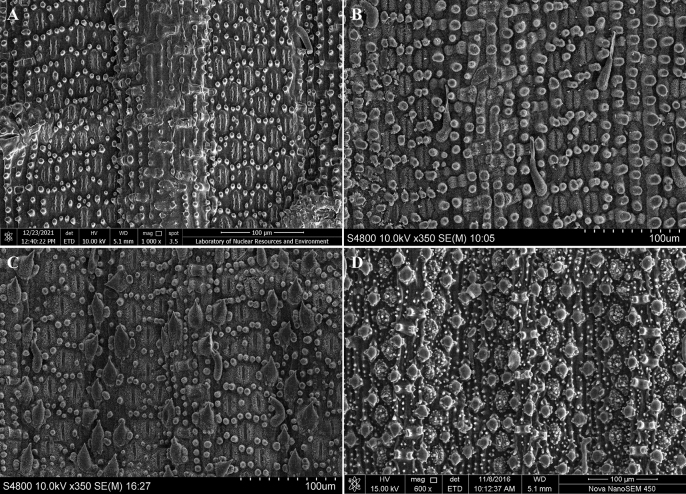
SEM images of the abaxial leaf epidermis **A***G.albozonatus* (Pengshui, Chongqing, China) **B***G.tessellatus* (Libo, Guizhou, China) **C***G.annulatus* (Chishui, Guizhou, China) **D***G.monophyllus* (Ningyuan, Hunan, China).

### ﻿Key to nine “gelido-” taxa of the genus *Gelidocalamus*

**Table:** 

1	Culm internodes glabrous	**2**
–	Culm internodes hairy	**5**
2	Culm leaf sheaths glabrous	**3**
–	Culm leaf sheaths pubescent with sparse setae	***Gelidocalamuszixingensis***
3	Culm sheaths glabrous; oral setae of culm leaves 1–2 pairs, weak; branch sheath margins hairless	***Gelidocalamusstellatus***
–	Culm sheaths covered with setae; oral setae of culm leaves 3–5 pairs; branch sheath margins with ciliate	**4**
4	Culm sheaths base covered with densely purple verrucous setulose	***Gelidocalamusalbozonatus***
–	Culm sheaths base glabrous	***Gelidocalamusmultifolius***
5	Culm sheaths with densely brown short setae	**5**
–	Culm sheaths with white erect small setae	**7**
6	Culms up to 5m tall, greater than 1cm in diam	***Gelidocalamustessellatus***
–	Culms less than 4m, less than 1cm in diam	**6**
7	Culm sheaths with white villus, margins with ciliate	***Gelidocalamusmonophyllus***
–	Culm sheaths hairless, margins hairless	***Gelidocalamusxunwuensis***
8	Culm sheath margins densely ciliate, oral setae 1 pair; leaves 1(or 2) per ultimate branch, lateral veins 6–9 pairs	***Gelidocalamuslatifolius***
–	Culm sheath margins hairless, oral setae 2–3 pairs; leaves 1–3 per ultimate branch, lateral veins 4–6 pairs	***Gelidocalamusannulatus***

## Supplementary Material

XML Treatment for Gelidocalamus albozonatus

## ﻿Additional information

### Author ORCIDs

Yong-Long Li https://orcid.org/0009-0004-1625-0140

Rong Guo https://orcid.org/0000-0001-6973-2734

Hong-Jing Zhang https://orcid.org/0000-0003-0025-9269

Si-Rong Yi https://orcid.org/0000-0002-3883-6175

Guang-Yao Yang https://orcid.org/0000-0002-6222-2130

Wen-Gen Zhang https://orcid.org/0000-0003-0946-8614

### Data availability

All of the data that support the findings of this study are available in the main text.
